# An enhanced electrochemical and cycling properties of novel boronic Ionic liquid based ternary gel polymer electrolytes for rechargeable Li/LiCoO_2_ cells

**DOI:** 10.1038/s41598-017-11614-1

**Published:** 2017-09-11

**Authors:** K. Karuppasamy, Hyun-Seok Kim, Dongkyu Kim, Dhanasekaran Vikraman, K. Prasanna, A. Kathalingam, Ramakant Sharma, Hee Woo Rhee

**Affiliations:** 1 Division of Electronics and Electrical Engineering, Dongguk University-Seoul, Seoul, 04620 South Korea; 2 Polymer Materials Lab, Department of Chemical and Biomolecular Engineering, Sogang University, 35 Baekbeom-ro,Mapo-gu, Seoul, 04107 South Korea; 3 Electrochemical Energy Storage and Conversion Lab (EESC), Kyung Hee University, 1732, Deogyeong-daero, Giheung-gu, Yongin, Gyeonggi 17104 South Korea; 4 Millimeter-wave Innovation Technology (MINT) Research Center, Dongguk University-Seoul, Seoul, 04620 South Korea; 5 Plastic Electronics and Energy Laboratory, Department of Metallurgical Engineering and Materials Science, Indian Institute of Technology Bombay, Powai, 400 076 Maharastra India

## Abstract

A new generation of boronic ionic liquid namely 1-ethyl-3-methylimidazolium difluoro(oxalate)borate (EMImDFOB) was synthesized by metathesis reaction between 1-ethyl-3-methylimiazolium bromide and lithium difluoro(oxalate)borate (LiDFOB). Ternary gel polymer electrolyte membranes were prepared using electrolyte mixture EMImDFOB/LiDFOB with poly vinylidenefluoride-*co*-hexafluoropropylene (*PVdF-co-HFP*) as a host matrix by facile solvent-casting method and plausibly demonstrated its feasibility to use in lithium ion batteries. Amongst ternary gel electrolyte membrane, DFOB-GPE3, which contained 80 wt% of EMImDFOB/LiDFOB and 20 wt% *PVdF-co-HFP*, showed excellent electrochemical and cycling behaviors. The highest ionic conductivity was found to be 10^−3^ Scm^−1^ at 378 K. Charge-discharge profile of Li/DFOB-GPE3/LiCoO_2_ coin cell displayed a maximum discharge capacity of 148.4 mAhg^−1^ at *C*/10 rate with impressive capacity retention capability and columbic efficiency at 298 K.

## Introduction

In the recent times, rechargeable lithium ion batteries are widely considered as one of the efficient electro-chemical energy storage system with high energy densities and therefore has been employed in many applications such as portable electronic devices, electric vehicles and smart grid storage systems^1–7^. However, several shortcomings which not only includes volatility and combustion of organic liquid electrolytes but also associated narrow operational temperature range restricts the frequent use of organic liquid electrolytes based lithium ion batteries^8, 9^. Thus, there is an urgent need to address all the above mentioned shortcomings so as to improve their operational stability and using them in hybrid electric vehicles^4, 5^. An ideal way to improve operational safety without compromising on the energy density of the batteries is to use gel polymer electrolytes (GPEs)^10–12^. Previous studies have demonstrated that GPEs have higher acceptable ionic conductivity than solid electrolytes at ambient temperature and higher thermal and mechanical stability than liquid electrolytes, making them a potential alternatives to all solid-state polymer electrolytes and traditional liquid electrolytes^9^. Till date various polymers such as poly (ethyleneoxide) (PEO), poly (propyleneoxide) (PPO), poly (methylmethacrylate) (PMMA), poly (acrylonitrile) (PAN), poly (vinylidene fluoride) (PVdF) and poly (vinylidene fluoride-co-hexafluoropropylene) (*PVdF-co-HFP*) have been introduced as hosts for GPEs preparations^13–16^. Amongst them, *PVdF-co-HFP* is considered as a better polymer host because of its high electrochemical stability and ability to dissolve lithium salts.

Usually organic solvents are having meager thermal and electrochemical properties and relatively narrow electrochemical potential window^17^. To address the aforementioned issues, scientific communities have been developed various types of additives to enhance electrochemical and cycling stability greater to organic solvents. On the other hand, room temperature ionic liquids (ILs) which consists of anions and cations have some exceptional properties such as negligible vapor pressure, non flammability, better thermal stability, great chemical and electrochemical stability, inherent long life, and high ionic conductivity are considered to be a promising electrolyte salt for polymer in salt system^18–22^. GPEs combines with ionic liquids which are phenomenally called ionic liquid gel polymer electrolytes (ILGPEs) possess the merits of high ionic conductivity, great electrochemical stability window and good charge-discharge performance by the way of preventing the dendrite formation on lithium metal electrodes. Henceforth, it is believed to be prospective electrolytes for LIBs. Remarkable efforts have been devoted to enhance the ionic conductivity and performance of GPEs for LIBs. Interestingly, different types of ionic liquids such as TFSI^−^, CF_3_SO_3_^−^, BF_4_^−^, C_4_F_9_SO_3_^−^ impregnated with lithium salts as electrolyte mixtures were successfully employed as GPEs for lithium-ion battery and the performance of those electrolytes and their batteries have extensively explored^18–23^.

The present work is focused on preparing a new generation of GPEs which comprised of boron anionic ionic liquid and lithium salt so as to fabricate advanced GPE with excellent mechanical strength, enlarged electrochemical window and high ionic conductivity. Here, we report an innovative ternary gel polymer electrolyte (TGPEs) membranes which are prepared by incorporation of diflurooxalato borate (DFOB) anion based ionic liquid [1-ethyl-3-methylimidazolium diflurooxalato borate (EMImDFOB)] and lithium salt (LiDFOB) into a *PVdF-co-HFP* matrix. Difluorooxalato borate (DFOB) is of quite interest in the present investigation due to its improved solubility compared with previously studied anions^24^. Also, in DFOB, the presence of more electron-withdrawing fluorine atom results into more delocalized charge which gives the anion less affinity for Li^+^ and EMIm^+^, causing better conductivity^25^. Moreover, it possesses a lower lowest unoccupied molecular orbital (LUMO) and a higher electrochemical stability for wide electrochemical window towards batteries with high energy. The detailed investigation is carried out to synthesis and calibration of EMImDFOB with LiDFOB for LIBs. The synergistic effect of EMImDFOB/LiDFOB on the electrochemical, thermal, mechanical and cycling performance of difluorooxalato borate based gel polymer electrolytes (denoted as DFOB-GPEs) have been demonstrated plausibly. To the best of our knowledge, there is no report available based on *PVdF-co-HFP*-EMImDFOB/LiDFOB as electrochemically and mechanically stable gel polymer electrolytes for LIBs applications.

## Results and Discussions

The synthetic route of ionic liquid is schematically represented in Fig. S1 (supplementary information). To demonstrate the purity of our synthesized ionic liquid EMImDFOB,^1^H NMR and ^19^F NMR analyses were carried out. The^1^H NMR (D_2_O as solvent) (*δ* scale related to TMS) spectrum of EMImDFOB shows important chemical shifts signals at 3.87 ppm (3 H, s, N-CH_3_), 8.68 ppm (1 H, s, N-CH-N), 7.45 ppm (1 H, d), 7.41 ppm (1 H, d), 4.22 ppm (2 H, q, NCH_2_) and 1.49 ppm (3 H, t, JZ1.8 Hz). Whereas its corresponding ^19^F-NMR (using D_2_O as solvent) spectrum depicts *δ* peak at 151.68 (2 F, t ^#^, J = 4.5 Hz, BF_2_) as shown in Fig. S2a,b) of supplementary information. The exhibited water content of as synthesized EMImDFOB is less than 35 ppm which is inferred from Karl-Fischer titration and this value is quite acceptable for practical lithium ion battery applications. The viscosity of EMImDFOB is found to be 123 ± 1 *cP* as shown in Fig. S3.

**Figure 1:**
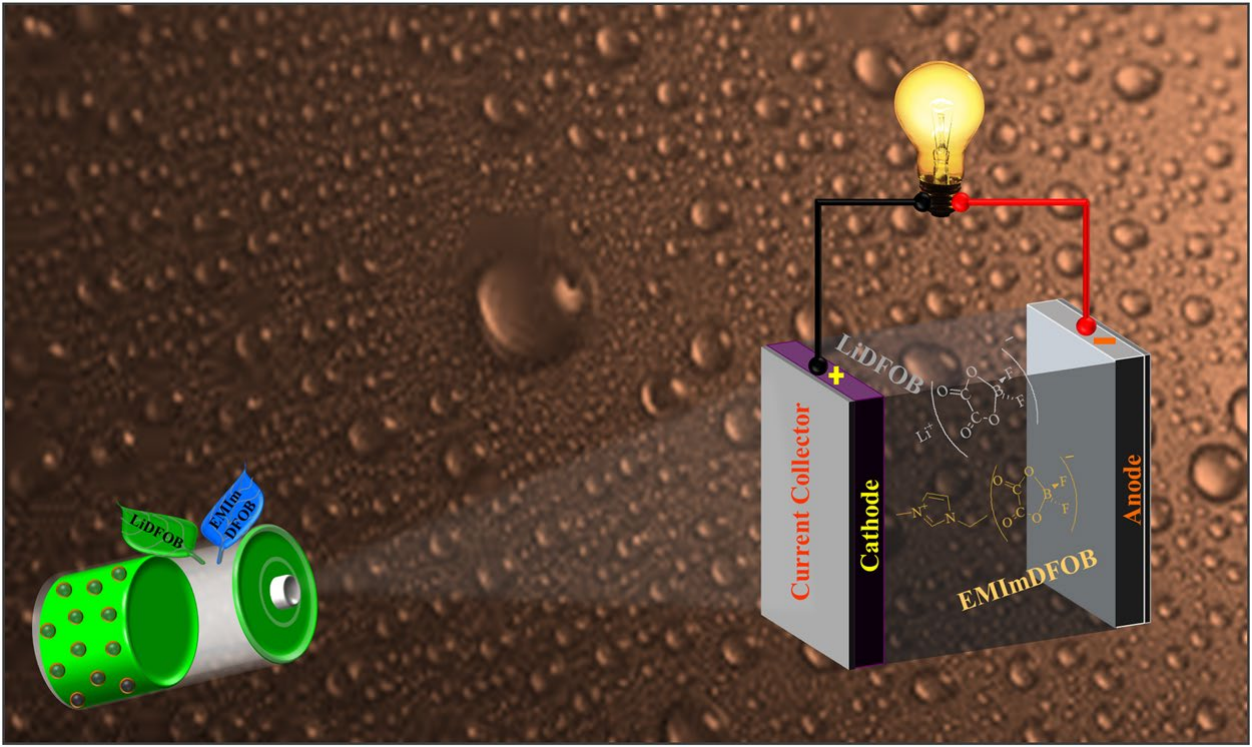
(**a**) The working module of LIBs using synthesized EMImDFOB/LiDFOB gel electrolytes.

**Figure 2:**
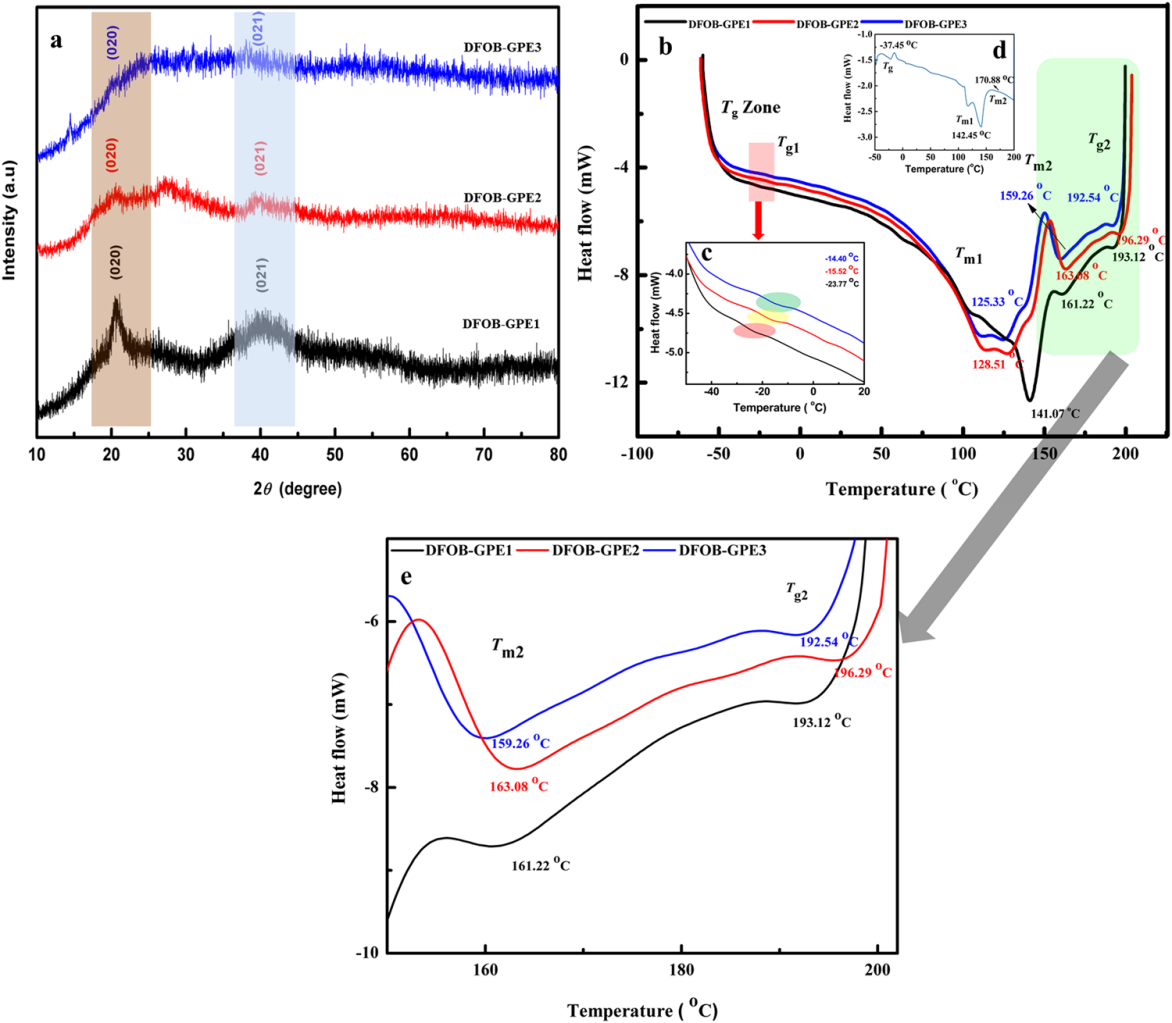
(**a**) XRD patterns of DFOB-GPEs; (**b**) DSC thermogram curves of DFOB-GPEs and inset figure shows (**c**) DSC thermogram of DFOBGPEs in the temperature region between −50 and 20 °C (**d**) DSC thermogram curve of pristine *PVdF-co-HFP* and (**e**) DSC thermogram of DFOBGPEs in the temperature region between 150 and 200 °C.

The innovative TGPEs membranes were prepared by incorporation of synthesized EMImDFOB and lithium salt LiDFOB into a *PVdF-co-HFP* matrix. The utilization of EMImDFOB/LiDFOB based ternary GPEs in LIBs are schematically represented in Fig. 1(a). XRD measurements were performed for pure *PVdF-co-HFP*, DFOB-GPE1, DFOB-GPE2 and DFOB-GPE3 to examine its phase purity and crystalline behavior. The XRD patterns of prepared DFOB-GPEs with different ratios are given in Fig. 2(a). For the comparison, pure *PVdF-co-HFP* XRD pattern is provided in Fig. S4. Two kind of characteristic diffraction peaks located at 2θ = 20.38° and 40.26° corresponds to (020) and (021) reflection planes, respectively, which revealed the semi-crystalline PVdF is presented in the complexes^26, 27^. Due to addition of EMImDFOB/LiDFOB into the *PVdF-co-HFP* matrix, it has been observed that the intensity of *PVdF-co-HFP* characteristic peak is decreased thereby confirming the decrease in crystalline behavior in the as prepared membranes. Furthermore, no other characteristic peaks are observed related to EMImDFOB/LiDFOB which affirms the complete dissolution of electrolyte mixture into the polymer matrix. The observed amorphous nature of XRD patterns (Fig. 2a) certifies the formation of DFOB-GPEs through the addition of EMImDFOB/LiDFOB into *PVdF-co-HFP* matrix system. In addition, XRD pattern reveals the absence of characteristic peak of *PVdF-co-HFP* for DFOB-GPE3 electrolyte, which is in agreement with the result by Qing Zhang *et al*.^27^.

Differential scanning calorimetry (DSC) analysis was performed to validate the XRD results as well as to identify the influence of EMImFOB/LiDFOB on *T*_g_ and *T*_m_ in *PVdF-co-HFP* matrix. Figure 2(b) shows the DSC thermograms of DFOB-GPE systems such as DFOB-GPE1, DFOB-GPE2 and DFOB-GPE3 with the temperature range between −70 and 200 °C. The DSC thermogram of pure EMImDFOB and EMImDFOB/LiDFOB are represented in Fig. S5. From the inset thermogram (Fig. 2d), an endothermic peak is appeared at around 142.25 °C corresponding to the melting of crystalline phase and the glass transition temperature (*T*_g_) peak is observed at −37.45 °C for *PVdF-co-HFP*. Whereas Tg values of pure EMImDFOB and EMImDFOB/LiDFOB are appeared around 186 and 197 °C respectively (Fig. S5). Enlarged spectra of inset Fig. 2(c) for region −45 to −20 °C revealed that the DFOB-GPEs system *T*_*g*_ zone is shifted from −23.77 to −14.40 °C with the increase in the EMImDFOB/LiDFOB concentration from 60 to 80 wt%, respectively. The incorporation of EMImDFOB/LiDFOB with polymer matrix elevates the *T*_*g*_ of DFOB-GPEs as well as widens the *T*_g_ zone Fig. 2(c and e). The *T*_g_ zone widening can be directly related to enhancement of flexibility in the polymer chains of ionic-polymer complex. Also, the melting temperature (*T*_m_) of our prepared DFOB-GPEs membranes is shifted towards lower temperature region by the addition of EMImDFOB/LiDFOB into *PVdF-co-HFP* matrix. This can be due to plasticization effect i.e., the presence of EMImDFOB/LiDFOB mixture in *PVdF-co-HFP* matrix might have weakened the interactive bonds between the chains within the gel electrolytes thereby causing the reduction in the amount of energy needed to break the bond. Amongst the prepared DFOB-GPEs, the gel electrolyte with 80% EMImDFOB/LiDFOB shows the lowest value of *T*_m1_ and *T*_m2_ as can be seen in Fig. 2(b and e) that the *T*_*m*_ of pure host (*T*_m1_ and *T*_m2_ @ 142.00, 170.88 °C) is decreased to 125.33 and 159.26 °C for DFOB-GPE3. The observed results are in concurrence with other ILGPEs using *PVdF-co-HFP* as host matrix^28, 29^. The observed *T*_g_, *T*_m_, *ΔH*_m_ and percentage of crystallinity (%α) values are tabulated in Table 1. The %α of DFOB-GPEs is calculated by following equation,

1 $$ \% \alpha =({\rm{\Delta }}{H}_{{\rm{m}}}/{\rm{\Delta }}{H}_{{\rm{m}}100 \% })\times 100$$

**Table 1:** The various thermal, electrochemical and mechanical parameters of DFOB-GPEs.

DFOB-GPEs	T_m_ (°C)		T_g_ (°C)		ΔH_m_ (J/cal)	% α	σ_298K_ (Scm^−1^)	E_a_ (eV)	Cut-off voltage (V)	Mechanical Strength (MPa)
	T_m1_	T_m2_	T_g1_	T_g2_						
DFOB-GPE1	141.07	161.58	−23.77	193.12	18.10	17.23	2.34 × 10^−6^	0.3717	4.04	4.2
DFOB-GPE2	128.51	161.15	−15.52	196.29	14.67	14.04	3.81 × 10^−5^	0.3197	4.24	2.8
DFOB-GPE3	125.33	159.56	−14.40	192.54	12.66	12.12	3.30 × 10^−4^	0.2816	4.46	2.1

DFOB-GPE1 (60% LiDFOB/EMImDFOB + 40% PVdF-HFP), DFOB-GPE2 (70% LiDFOB/EMImDFOB + 30% PVdF-HFP) and DFOB-GPE3 (80% LiDFOB/EMImDFOB + 20% PVdF-HFP)

T_g_ values for pure EMImDFOB & EMImDFOB/LiDFOB are 186 and 197 ^o^C respectively (Fig. S5).

The heat of enthalpy (${\rm{\Delta }}{H}_{m100 \% }$) value is observed at 104.5 J/g for 100% crystalline *PVdF*-*co*-*HFP* ^14^. The value of %α is decreased from 17.23 to 12.12 (Table 1) with increase of EMImDFOB/LiDFOB content in DFOB-GPEs which revealed the flexibility and mobility enhancement in the polymer chain segment of gel electrolytes.

**Figure 3:**
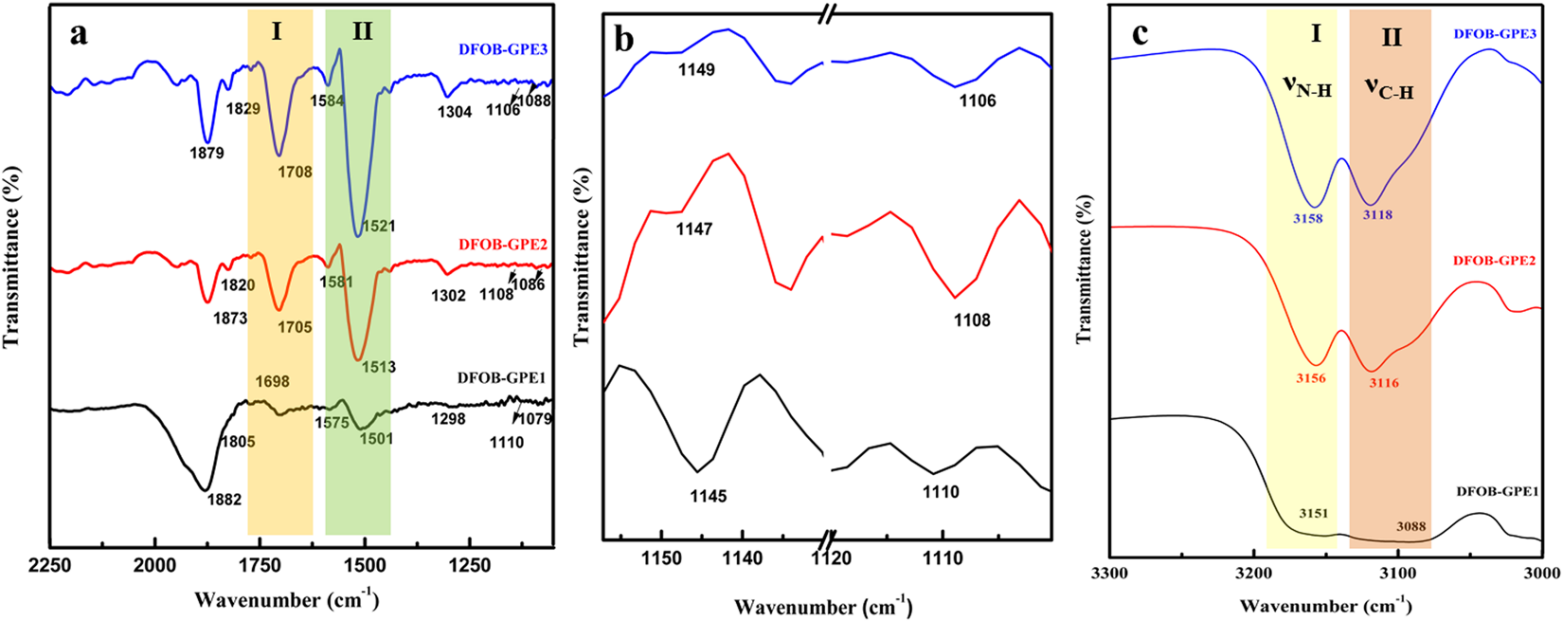
FT-IR spectra of DFOB-GPEs in the wavenumber regions (**a**) 2250–1200 cm^−1^ (The highlighted regions I and II represents *symmetrical ν*_*C*=*O*_ *and vibrational δ*_*CH*_ *of imidazolium ring* (region II) for DFOB-GPEs (**b**) 1160–1100 cm^−1^ and (**c**) 3300–3000 cm^−1^(The highlighted regions I and II represents and s*ymmetrical ν*_*N-H*_ (region I) *and ν*_*C-H*_ of imidazolium ring (region II) for DFOB-GPEs.

To explore the significance of EMImDFOB/LiDFOB with *PVdF-co-HFP* system, the attenuated total reflection (ATR) – Fourier transform-infrared (FTIR) spectroscopy analysis was performed for our DFOB-GPEs membranes. Figure 3(a) represents the ATR-FTIR spectra of DFOB-GPE1, DFOB-GPE2 and DFOB-GPE3 polymer membranes. The FTIR peaks appeared at 1120 cm^−1^ and 1040 cm^−1^ belongs to asymmetrical stretch of CF_2_ group and bending of CF_3_ group of pure *PVdF-co-HFP*, respectively^11, 30, 31^. For pure EMImDFOB/LiDFOB mixture (Fig. S6), FTIR characteristic vibrational peaks observed at 1800, 1625, 1362, 1298, 1180, 1085 and 822 cm^−1^ corresponds to the symmetrical C=O stretching, asymmetrical C=O stretching, asymmetrical C-O-C stretching, O-B-O bending, O-B-O symmetrical stretching, B-F symmetrical stretching and B-F bending, respectively^32, 33^. The addition of EMImDFOB/LiDFOB in *PVdF-co-HFP* matrix facilitates the interaction with the free electron pairs of fluorine atom (CF_2_ and CF_3_ group) of host matrix as can be confirmed by low intensity vibrational peaks in the region of 1100–1175 cm^−1^ as represented in Fig. 3(b). Also, the shift of ν_a_(CF_2_) and δ(CF_3_) vibrational modes towards higher wavenumber reveals the cations (Li^+^ and EMIm^+^) interaction with the polymer host. Moreover, the formation of complex of EMImDFOB/LiDFOB with *PVdF-co-HFP* matrix is confirmed by important conformational mode variations in two regions namely region I (1740–1620 cm^−1^) and region II (1570–1480 cm^−1^) as highlighted in Fig. 3(a). Further, the formation of complex of EMImDFOB/LiDFOB with *PVdF-co-HFP* is evident by the shifting of ν_*CH*_ and ν_*NH*_ peaks towards right in the wavenumber region 3300–3000 cm^−1^ as shown in Fig. 3(c). The particular attentiveness for these two regions is due to some crucial changes in the peak positions which are given in following. **(a)** ***Region I*** - ***Symmetrical ν***_***C***=***O***_ ***of DFOB*** **:** The deconvoluted spectra of DFOB-GPEs for region-I (1740–1620 cm^−1^) is shown in Fig. S7. For DFOB-GPE1, the symmetrical stretching ν_*C=O*_ bands were appeared at 1702 and 1687 cm^−1^ which can be assigned to ion pairs and free ions, respectively with low peak broadening. The broadened ν_*C=O*_ bands were observed at 1705 and 1649 cm^–1^ for DFOB-GPE2 and at 1705 and 1644 cm^−1^ for DFOB-GPE3 which are related to the ion pairs and free ions, respectively. The observed results are consistent with the earlier report^34–36^. **(b)** ***Region II*** - ***δ***_***CH***_ ***and ν***_***CH***_ ***of imidazolium ring:*** In the region II of Fig. 3a (1570~1480 cm^−1^) and 3c (3300–3000 cm^−1^), all the DFOB-GPEs exhibits the vibrational bands which belongs to δ_CH_ and ν_*CH*_ of imidazolium ring. The characteristic vibrational peak of DFOB-GPE became prominent for the higher concentration of EMImFOB/LiDFOB and also vibrational peak of pure polymer host tends to disappear which indicates that presence of EMImFOB/LiDFOB is crucial in the gel electrolyte matrix system and it has increased the amorphicity^29, 37^.

**Figure 4:**
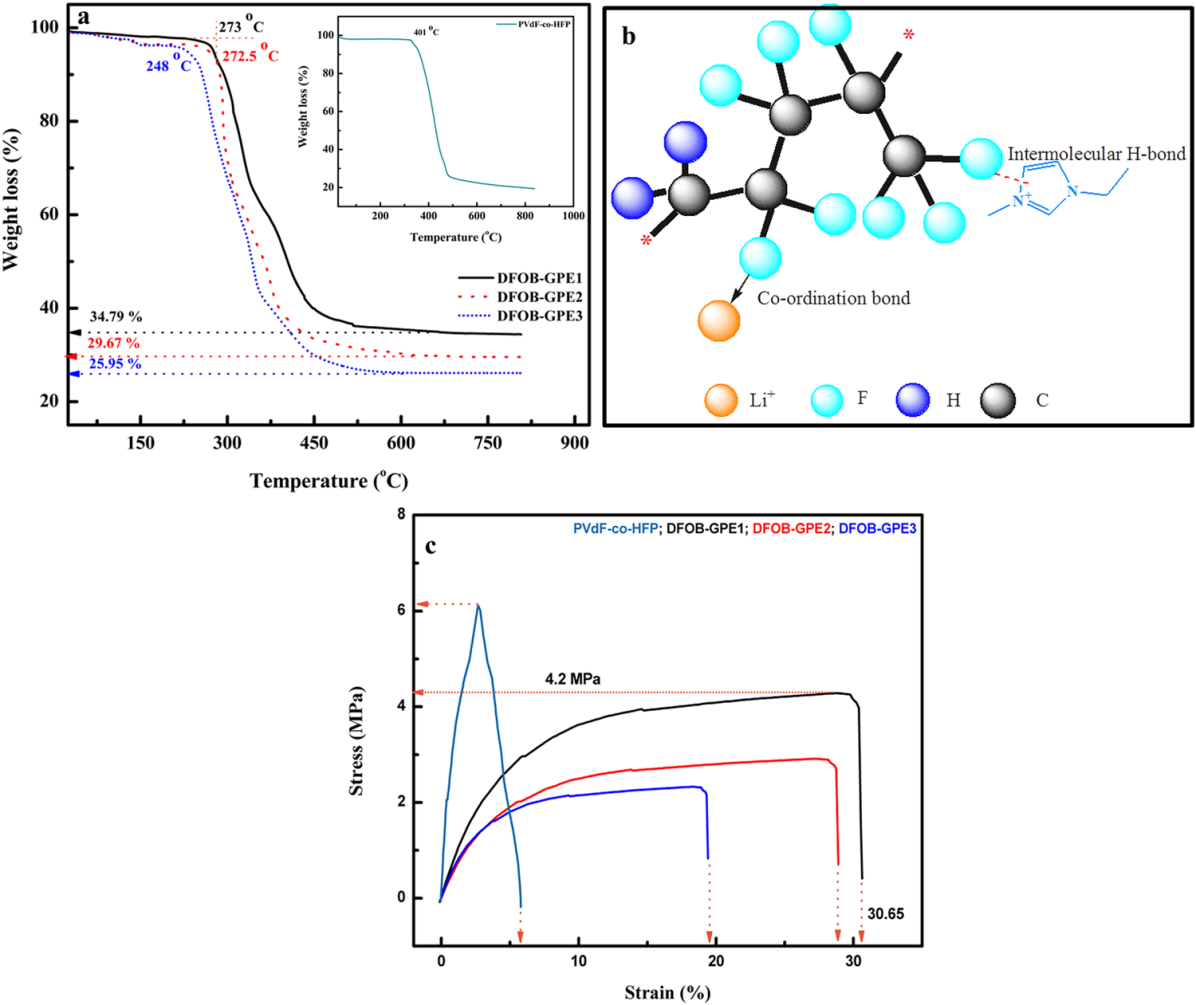
(**a**) TGA thermogram of DFOB-GPEs (Inset – TGA curve for pristine *PVdF-co-HFP*); (**b**) Possible intermolecular mechanism between *PVdF-co-HFP* and EMImDFOB/LiDFOB in DFOB-GPEs (**c**) Stress strain curves of pristine *PVdF-co-HFP* and DFOB-GPEs.

TGA analysis was performed to verify the thermal decomposition of prepared DFOB-GPEs and to analyze its dimensional stability at elevated temperatures. The TGA plots of DFOB-GPE1, DFOB-GPE2 and DFOB-GPE3 in the temperature range of 25–800 °C are shown in Fig. 4(a). The TGA plot of pristine *PVdF-co-HFP* is given in inset of Fig. 4(a) whereas the TGA plot for pure EMImDFOB and EMImDFOB/LiDFOB are shown in Fig. S8. It is observed that the decomposition of all DFOB-GPEs is single step and decomposition temperatures of various DFOB-GPEs are found to be 273, 272 and 248 °C respectively. From Fig. 4(a), it can be found that for the higher content of EMImDFOB/LiDFOB based DFOB-GPEs (DFOB-GPE3), the thermal stability decreases slightly (273 °C to 248 °C) but is still suitable for practical application. The decrease in thermal stability at high electrolyte mixture (EMImDFOB/LiDFOB) content is explained as follows: After incorporation of EMImFOB/LiDFOB into *PVdF-co-HFP*, the onset of thermal decomposition temperature for DFOB-GPEs decreases slightly, which reveals that the existence of interaction between *PVDF-co-HFP* and EMImFOB/LiDFOB. These interactions results from intermolecular hydrogen bonds between fluorine atoms and the hydrogen atoms connected with carbon atom in imidazole ring of EMImDFOB^38^ as represented pictorially in Fig. 4(b). Further, as it can be seen from the thermogram that the drastic weight losses of 34.79, 29.67 and 25.95 wt% were observed for DFOB-GPE1, DFOB-GPE2 and DFOB-GPE3, respectively. Amongst them, DFOB-GPE3 possesses remarkably low percentage of weight loss than that of other two electrolytes. To explain the quality of our result, it is worth mentioning that the previous studies by Tang *et al*. have reported weight loss of 75 wt% of *PVdF-co-HFP* based gel electrolyte systems which is higher than our gel electrolyte membrane^22, 38^.

Stress-strain behavior of gel polymer electrolytes plays a key role to determine the exact mechanical strength of prepared electrolytes as well as to prevent the short circuit in lithium ion batteries application. The typical stress-strain curves of pristine PVdF-*co* HFP, DFOB-GPE1, DFOB-GPE2 and DFOB-GPE3 are shown in Fig. 4(c). It can be seen from Fig. 4(c) that the pristine *PVdF-co-HFP* shows a one-step break (@6.09 MPa) mechanism resulting in non-linear elastic behavior with the strain of 5.77%. In the case of DFOB-GPEs, the two discrete regions are observed such as linear region for elastic characteristic and nonlinear region for plastic deformation. When increase the ratio of EMImDFOB/LiDFOB in DFOB-GPEs membranes, the mechanical strength is drastically decreased and flexibility is linearly enhanced. In Comparison with DFOB-GPE2 and DFOB-GPE3, the DFOB-GPE1 membrane which is containing 60% of ionic liquid EMImDFOB/LiDFOB has low elongation break (29.00%) and high tensile strength (2.93 MPa). For DFOB-GPE3, decrease in the tensile strength (2.22 MPa) and increase in strain at 34.72% is observed. From the above results of DFOB-GPEs, it can be inferred that DFOB-GPE3 possesses low mechanical strength compared to other electrolytes but it possess appreciable robustness (2.22 MPa), self-standing with no electrolyte flow, which can meet the requirement for practical lithium ion battery applications. This is in agreement with the earlier reports^39–43^.

**Figure 5:**
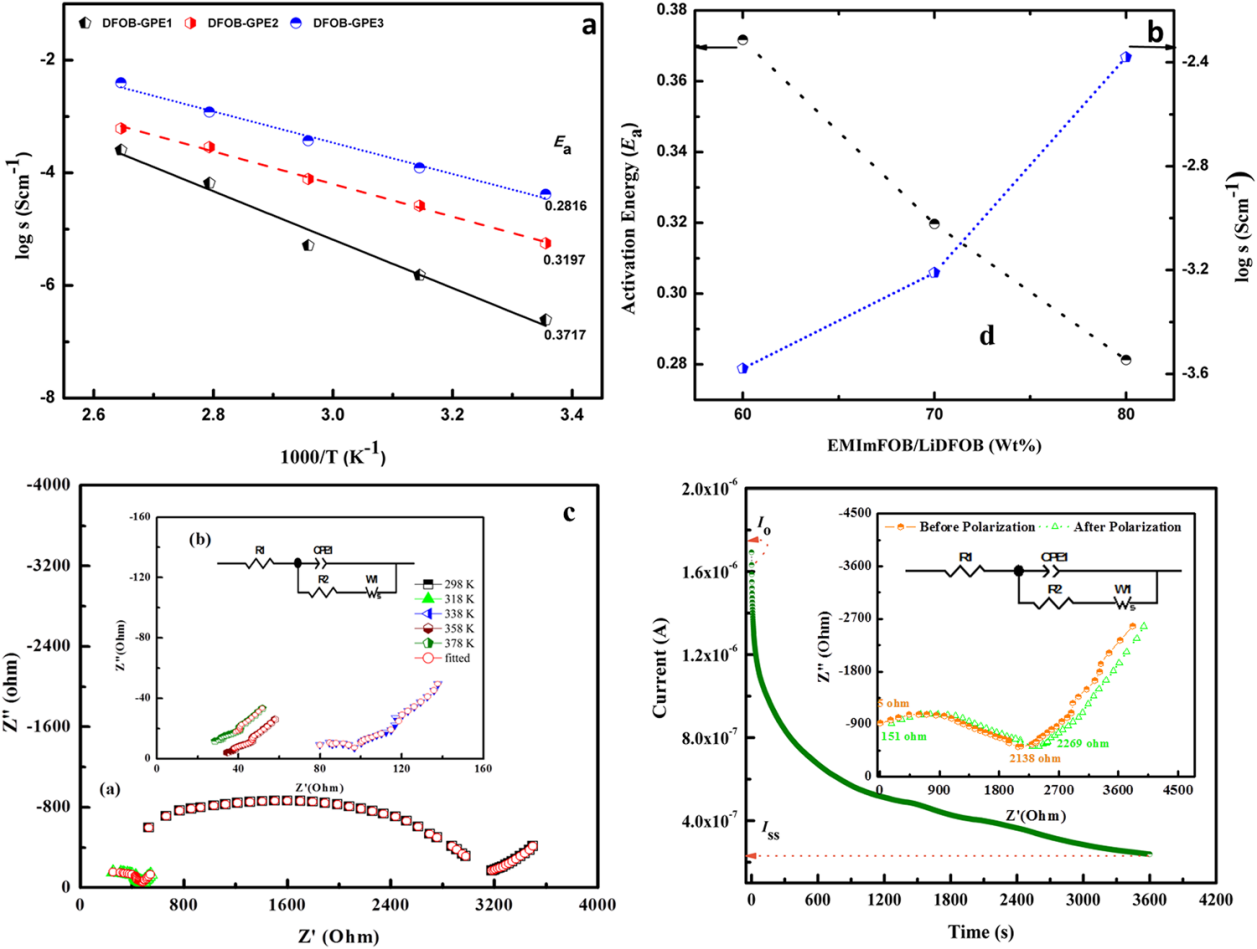
(**a**) Temperature dependence of Ionic conductivity for DFOB-GPE1, DFOB-GPE2 and DFOB-GPE3; (**b**) Comparison of activation energy and log σ_RT_ as function of electrolyte mixture content; (**c**) Nyquist impedance plot of DFOB-GPE3 and its corresponding equivalent circuit (inset); (**d**) Chronoamperometry curve of DFOB-GPE3.

In order to validate the electrochemical properties of electrolytes, we have performed electrochemical impedance spectroscopy (EIS) analysis for DFOB-GPEs in the temperature range between 298 and 398 K. The ionic conductivity as a function of temperature for all the DFOB-GPEs is presented in Fig. 5(a). It has revealed that the ionic conductivity increases with increase of temperature for all the DFOB-GPEs samples. Also, our observed result obeys Arrhenius law of conduction relation as follows

2 $$\sigma ={\sigma }_{0}{{\rm{e}}}^{-({E}_{a}/RT)}$$

where *σ* is the ionic conductivity, T is the absolute temperature, *E*_*a*_ is the apparent activation energy for ionic transport. *R* is the gas constant (8.314 J/mol.K). The calculated activation energy (*E*_*a*_) and pre-exponential factor (*σ*_0_) values are listed in Table 1. The values of *E*_*a*_ and *σ*_0_ of gel electrolytes decreases with increase in EMImDFOB/LiDFOB weight percentage (wt. %) in DFOB-GPEs as shown in Fig. 5(b). Also using the above equation (2), the calculated values of conductivity at 298 K for DFOB-GPE1, DFOB-GPE2 and DFOB-GPE3 is found to be at 2.34 × 10^−6^, 3.81 × 10^−5^ and 3.30 × 10^−4^ Scm^−1^, respectively. At high temperatures, all DFOB-GPEs demonstrates the increase in the conductivity thereby yielding the values of 1.80 × 10^−4^, 6.13 × 10^−4^ and 4.11 × 10^−3^ Scm^−1^ for DFOB-GPE1, DFOB-GPE2 and DFOB-GPE3, respectively. The Nyquist plot with equivalent circuit for DFOB-GPE3 is shown in Fig. 5(c) with different temperatures and its corresponding equivalent circuit parameter values are tabluted in Table S1. The observed high ionic conductivity of DFOB-GPE3 can be attributed to the faster migration of charge carriers in polymer matrix with high flexibility when compared to other two electrolytes. Previous report by Stepniak *et al*.^44^ have demonstrated that conductivity of gel electrolytes is ~0.64 × 10^–4^ Scm^−1^ which is lower than observed results for all the DFOB-GPEs. Also, they have stated that the ionic conductivity of electrolyte membrane depends on the number of charge carriers as it act as a medium between anode and cathode as well as served as a host for the whole system. Amongst all prepared DFOB-GPEs, DFOB-GPE3 provides highest ionic conductivities of the order of 10^−4^ Scm^−1^ and 10^−3^ Scm^−1^ at room temperature and 398 K, respectively. The resultant conductivity values are quite higher than that of previously studied *PVdF-co-HFP* based gel electrolyte system^11, 44–46^ which makes our prepared DFOB-GPEs as a potential candidate for lithium ion batteries with wide range of operating temperatures.

To explore the role of lithium ions as well as transfer rate of lithium ions in the electrolyte, we have demonstrated cationic transference number using chronoamperometry and EIS analyses. The symmetrical Li/DFOB-GPE3/Li was conducted at room temperature and the resulting polarization curve of DFOB-GPE3 is shown in Fig. 5(d). We have used DFOB-GPE3 electrolyte for analyses because it possesses the maximum ionic conductivity than that of presented other gel electrolyte systems. The lithium transference number (*t*_Li_^+^) of DFOB-GPEs was obtained by equation (3)

3 $${{\rm{t}}}_{L{i}^{+}}={I}_{{\rm{s}}{\rm{s}}}({\rm{\Delta }}V-{R}_{0}{I}_{0})/{I}_{0}({\rm{\Delta }}V-{R}_{{\rm{s}}{\rm{s}}}{I}_{{\rm{s}}{\rm{s}}})$$

where, the subscripts 0 and ss represents initial values and steady state values, respectively, *R*_b_ is the bulk resistance, *R* is the passive film resistance, and their values can be evaluated from the nyquist curves of the DFOB-GPEs before and after the experiment, ${\rm{\Delta }}V$ is the applied voltage and *I* is the current. As evident from the Fig. 5(d), the chronoamperometric curve tends to decrease linearly with time for symmetric cell Li/DFOB-GPE3/Li. The value of *t*_Li_^+^ for DFOB-GPE3 is found to be at 0.37 which is quite high compared to other *PVdF-co-HFP* based systems^47^. The higher value of *t*_Li_^+^ is due to the faster migration of lithium ions in the gel matrix which can be explained by following facile mechanism: The cationic sites such as Li^+^ and EMIm^+^ can interact with the electron donor site of *PVdF-co-HFP* (CF_2_) and DFOB^−^ and therefore can possibly weakened the polymer backbone chain thereby increasing the amorphocity of gel electrolyte to yield high electrical properties. Due to the amorphous nature of polymer host matrix, the transport of charge carrier Li^+^ becomes faster thereby improving the cationic transport number. The observed results explains the increase in the ionic conductivity in DFOB-GPEs membranes as well as transference number which is due to the inclusion of EMImDFOB/LiDFOB in *PVdF-co-HFP* matrix system^48^.

**Fig. 6:**
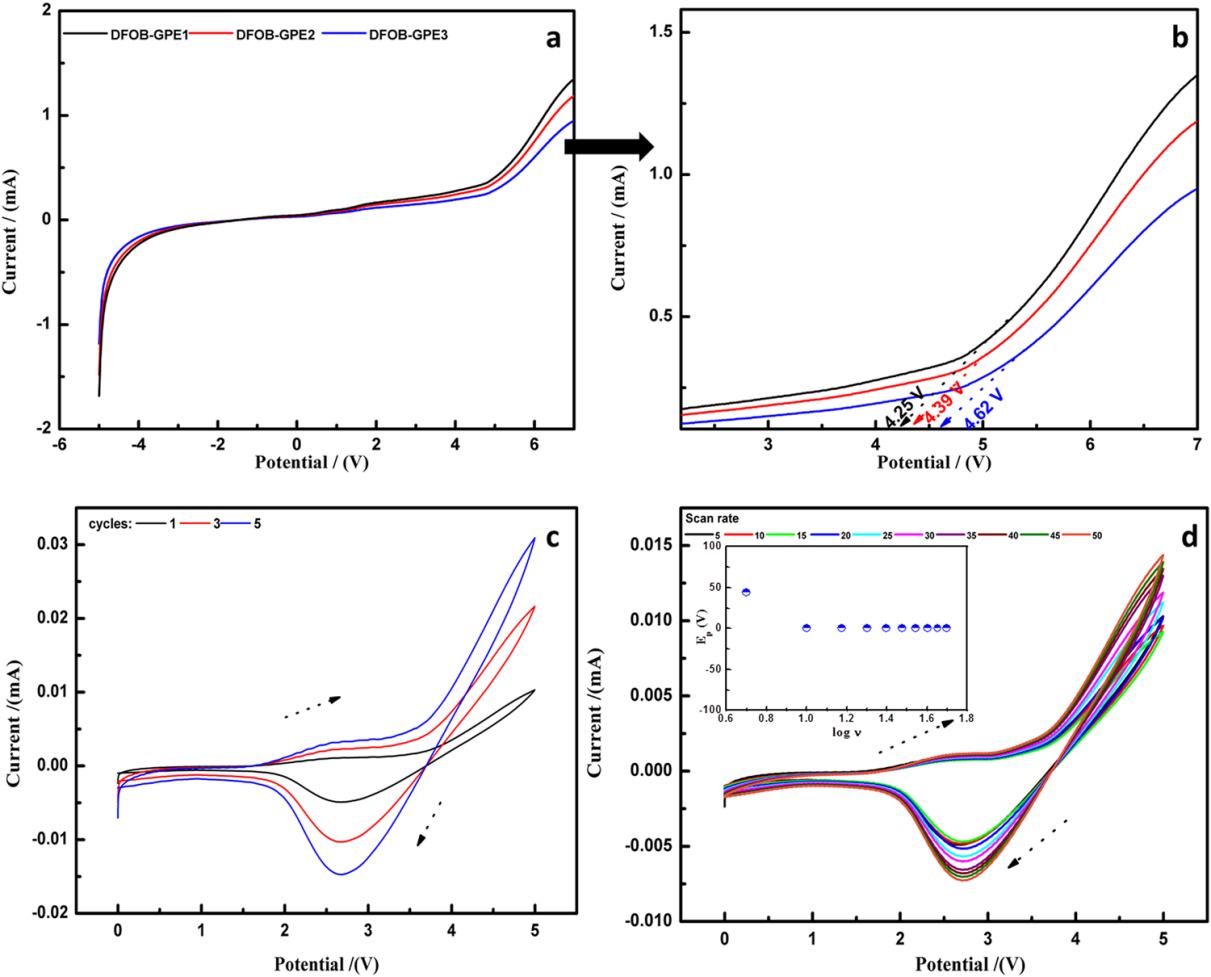
(**a**) linear sweep voltammogram of DFOB-GPE1, DFOB-GPE2 and DFOB-GPE3; **(b)** Cyclic voltammogram of high conducting DFOB-GPE3 at different cycles; (**c**) Cyclic voltammogram of high conducting DFOB-GPE3 at different scan rates (inset- Peak potential (E_p_) as a function of scan rates).

Linear sweep voltammetry (LSV) was performed in order to determine the operating potential and the electrochemical stability window of prepared electrolytes for batteries with the potential range between −5 and + 6 V. The LSV scans were performed using Pt/DFOB-GPEs/Li cell with a scan rate of 20 mV/s at 298 K. LSV curves of DFOB-GPEs membranes are shown in Fig. 6(a). From the spectra, LSV current (*I*) is increased with the applied voltage (*V*) for all the DFOB-GPEs membrane. The breakdown voltage of DFOB-GPEs increases with increase of EMImDFOB/LiDFOB contents and it is observed at 4.04, 4.24 and 4.46 V vs. Li/Li^+^ for DFOB-GPE1, DFOB-GPE2 and DFOB-GPE3, respectively_._ This obvious improvement of the breakdown voltage demonstrates that our DFOB-GPE3 may have potential advantage for applications in practical high performance lithium ion batteries^49, 50^.

Further, to validate LSV results as well as to confirm lithium ion conduction into the GPEs, cyclic voltammetric (CV) analysis was carried out using a Li/DFOB-GPE3/LiCoO_2_ coin cell. Among all the DFOB-GPE membrane, DFOB-GPE3 is of significant interest due to its high ionic conductivity in ambient temperature and thermal stability than other two electrolytes. Figure 6(b) shows the multiple cycle CV curves of DFOB-GPE3 membrane comprised coin cell. From the 1^st^ cycle of CV curve, the wide electrochemical stability voltage windows (5 V) is observed and it has displayed lithium stripping at 2.6 V and lithium plating at 2.7 V. Hence, anodic stripping and cathodic deposition are facile at electrode-electrolyte interface. The lithium stripping peak is observed at 2.6 V and the lithium plating peak appears at 2.7 V during anodic and cathodic scans which clearly indicates that the DFOB-GPE3 is electrochemically active. In order to confirm the red-ox behavior of DFOB-GPE3 system, five consecutive cycles of CVs (Fig. 6b) is recorded and their peak stability is established. The red-ox peaks current values are increased with increase of number of cycles which may be due to faster ionic transport rate in electrode-electrolyte interface^22, 51, 52^.

The scan rate dependence of the redox peak currents and the peak-to-peak separation (*E*_p_) of DFOB-GPE3 were also analyzed, and the results are depicted in Fig. 6(c). The scan rate was varied from 5 to 50 mV/s. It was noticed that with the increase of the scan rate, the oxidation peaks shifts towards higher potential while its counterpart peak shifts towards lower potential. The value of *E*_p_ is plotted versus the log of scan rate and is presented in inset of Fig. 6(c). According to Laviron’s theory^38^, with the increase of scan rate, the oxidation and reduction peak currents of DFOB-GPE3 increases significantly. The observed results are strongly paved a way to utilize DFOB-GPE3 as an active electrolyte for lithium ion batteries.

**Figure 7:**
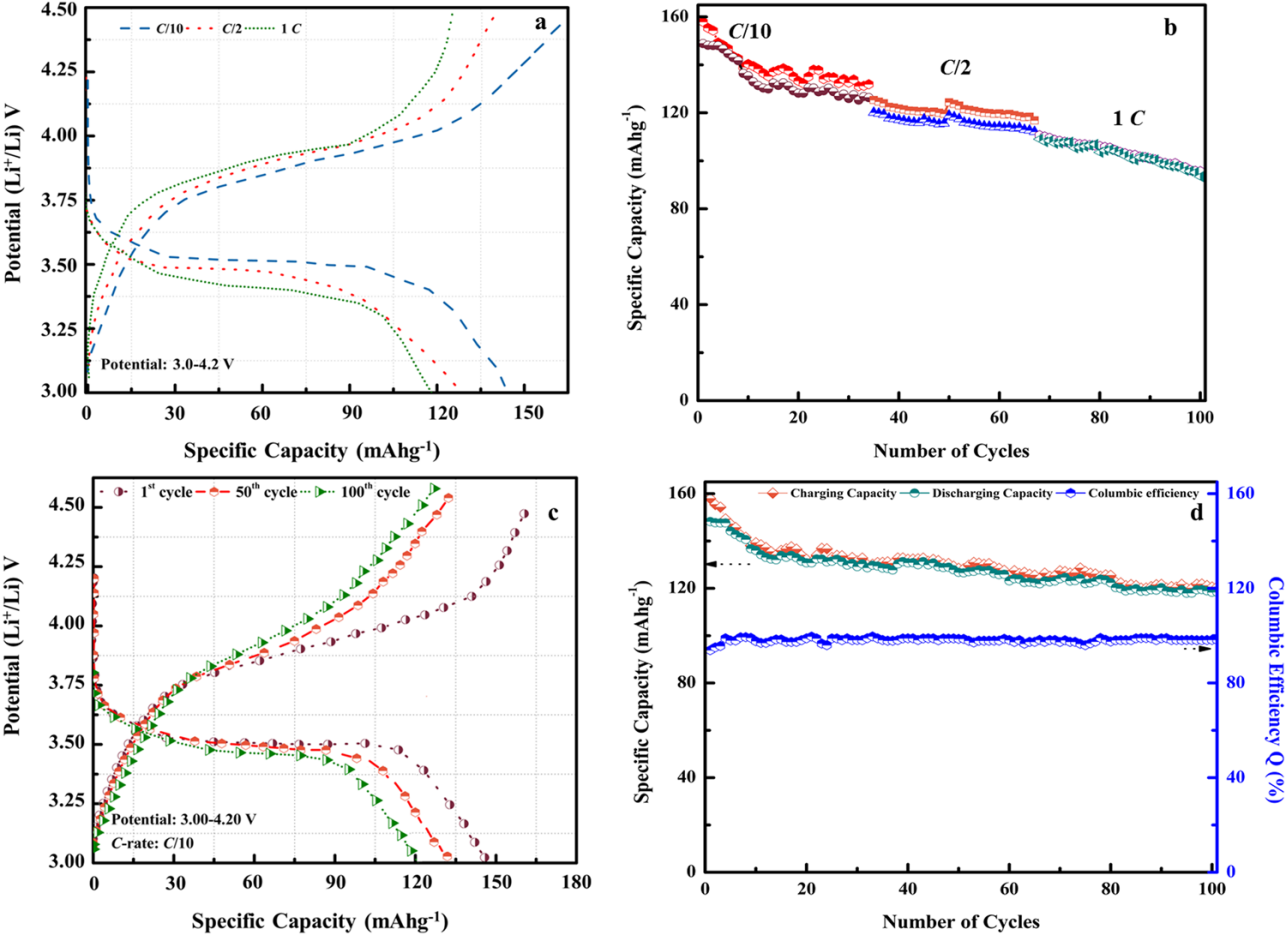
(**a**) Charge-discharge plateau of LiCoO_2_/DFOB-GPE3/Li cell at different *C*-rates; (**b**) Rate capability curve of LiCoO_2_/DFOB-GPE3/Li cell at different *C*-rates; (**c**) Charge-discharge capacities of LiCoO_2_/DFOB-GPE3/Li cell for (1^st^, 50^th^ and 100^th^) for 100 cycles at *C*/10 rate; (**d**) Columbic efficiency plot of LiCoO_2_/DFOB-GPE3/Li cell at C/10 *C*-rate.

With the consideration of mechanical and thermal stabilities, high ionic conductivity and transference number and better CV performance, the DFOB-GPE3 electrolyte was used to assemble a LiCoO_2_/Li coin cell. The discharge profiles are obtained at different current rates at room temperature as shown in Fig. 7(a). It seems that the discharge profile exhibits obvious stable voltage profiles at all the different C-rates which may be due to the better electrochemical stability and mechanical integrity of DFOB-GPE3. The discharge capacity slightly diminishes with number of cycles for different *C*-rates. The cycling performance at *C*/10 rate, with a discharge capacity of 148.4 mAh g^−1^, is apparently good. However, the discharge capacity faintly decreases to 130 and 118 mAh g^−1^ at *C*/2 and 1 *C* rates, respectively which is from 84 to 36% of the theoretical capacity for active material LiCoO_2_^53, 54^. The reversibility of the electrolyte is at 87, 54 and 40% for *C*/10, *C*/5 and 1 *C* rates, respectively. Its corresponding rate capability curve is displayed in Fig. 7(b). The outstanding performance of DFOB-GPE3 with different rates is ascribed to satisfactory performance of ionic conductivity and favorable electrochemical properties between the electrodes and the electrolyte in the cell.

Figure 7(c) represents the specific capacity as a function of number of cycles for LiCoO_2_/DFOB-GPE3/Li cell at *C*/10 rate. The cell delivers an initial discharge capacity of 148.4 mAhg^−1^ at first cycle performance. A slight decay in capacity occurs with increase of number of cycles and it delivers a discharge capacity of 120 mAhg^−1^ after 100^th^ cycle. The slight decrease in capacity during the first few cycles may be due to formation of passive layers over the lithium electrode surface during the cycling^55–58^. Moreover, no obvious changes are observed in charge capacity after few cycles which infers from the capacity retention curve as shown in Fig. 7(d). The columbic efficiency of the first cycle is merely 93.8% which increases gradually with number of cycles and observed at 98.2% for 100^th^ cycle. These findings imply that the DFOB-GPE3 possesses high specific capacity with very good capacity retention at ambient temperature and would be highly suitable with lithium anode and LiCoO_2_ cathode for lithium ion battery applications.

## Conclusions

In this paper, we have demonstrated the design and fabrication of novel ternary gel polymer electrolytes comprising of borate anion (DFOB) based ionic liquid and lithium salt with high electrochemical and cycling stability. The interactions between the ions and polymer were demonstrated by IR shifting of the ν_C=O_ mode for electrolyte mixture as well as the ν_a_(CF_2_) mode for *PVdF-co-HFP*. As obtained DFOB-GPEs have very high thermal stability with appropriate mechanical strength. The DFOB-GPE3 which comprises 80% LiDFOB/EMImDFOB and 20% *PVdF-co-HFP* has high ionic conductivity (10^−4^ Scm^−1^) and ionic transference number (0.37) at room temperature. The prepared LiCoO_2_/DFOBGPE3/Li cell possesses very good capacity retention and it provides a maximum of 148.4 mAhg^−1^ at *C*/10 rate. These outstanding properties of DFOB-GPEs pave a way to utilize as a potential candidate for application in future large scale rechargeable lithium ion batteries.

## Materials and Methods

### Source materials

Poly (vinylidinefluoride-co-hexafluoropropyline) (*PVdF-co-HFP* pellets, average molecular weight, *M*_w_ 4 × 10^5^), 1-ethyl 3-methyl imidazolium bromide (EMImBr, ≥99.1% pure, *M*_w_ 169.25), anhydrous solvents such as acetonitrile and dichloromethane were gifted from Sigma Aldrich. Lithium difluoro(oxalate)borate (LiDFOB, MW = 143.77 g/mol) were purchased from Suzhou Fosai New Material Co., Ltd., China. The lithium salt and other precursors were kept under *vacuum* at 50 and 70 °C, respectively for 12 h prior to use.

### Synthesis of 1-ethyl-3-methylimidazolium difluoro(oxalate) borate (EMImDFOB)

The EMImDFOB was synthesized by metathesis reaction between 1-ethyl-3-methylimidazolium bromide (EMImBr) and LiDFOB. In brief, 50 ml solution of EMImBr (0.4 M) was instinctively stirred for 4 h under nitrogen (N_2_) atmosphere at 60 °C. Then, 0.43 M solution of LiDFOB was added drop wise into stirred EMImBr solution. The obtained homogeneous solution was continuously stirred for 12 h at 60 °C under N_2_ atmosphere. The final product was extracted by rotatory with dichloromethane (DCM). After the DCM evaporation, it was further vacuum dried at 80 °C for 24 h and collected the yield of 58%. In the whole reaction process, double deionized water was used as a solvent. The as-prepared ionic liquid of EMImDFOB was stored in a glove box for further characterization. The excellent purity of the sample was further confirmed by ^1^H and ^19^F.

### Preparation of difluoro(oxalate)borate gel polymer electrolytes (DFOB-GPEs)

Firstly, to prepare electrolyte mixture of EMImDFOB/LiDFOB, 0.3 M of LiDFOB was dissolved in neat ionic liquid (EMImDFOB) and then mechanically stirred for 8 h at 60 °C under N_2_ atmosphere. The resultant solution mixture was degassed for about 15 min. The various ratios of polymer and electrolyte mixture (as shown in Table 1) were dissolved in anhydrous acetonitrile and constantly mixed for 8 h to get homogeneous viscous solution. Then different composition mixtures of EMImDFOB/LiDFOB and *PVdF-co-HFP* were prepared by solvent-casting method as reported earlier^22^. The whole process was carried out in an inert atmosphere. Surface of the obtained DFOB-GPEs films was constructed with copolymer network which consisting of EMImDFOB/LiDFOB as shown in Fig. S9. In order to avoid moisture effects, the prepared DFOB-GPEs films were stored in the glove box for further application and characterization analyses.

### Characterization techniques

^1^H-NMR and ^19^F-NMR spectra were taken on a Bruker avance 400 spectrometer. The water content of synthesized ionic liquid was determined by Karl-Fisher coulometric moisture titrator using a Mettler 831 KF autotitrator (Metrohm Co.,). Viscosity (*η*) measurement was performed using Modular compact rheometer (Physica MCR 301, Anton Paar).

The crystalline nature of synthesized gel electrolytes were examined by D-MAX 2500, Rigaku, X-ray diffractometer instrument equipped with Cu-Kα (λ = 1.5406 A°) radiation source, with a scan speed of 2° per minute, ranging from 0° to 100°. The differential scanning calorimetry (DSC) measurement of pure polymer host and DFOB-GPEs were made on TA instruments, (Model 2920) thermal analyzer at a heating rate of 10 °C min^−1^ under nitrogen atmosphere in the temperature range of −50 to 200 °C. The thermogravimetry (TGA) analyses of DFOB-GPEs were carried out using TGA 2950 (Hi-Res, TA instruments). The TG profiles were recorded under N_2_ flow (20 mL min^−1^), in a temperature range between 25 and 800 °C. A temperature ramp rate of 20 °C min^−1^ was used. FTIR spectra were recorded with the help of a Nicolet 380 FT-IR spectrometer (Thermo Electron) in the region 4000–400 cm^−1^ at a signal resolution of 1 cm^−1^. The mechanical stability of the gel electrolytes were measured using Instron Tester 6025 with computer evaluation. The ionic conductivities of prepared DFOB-GPEs were analyzed by electrochemical impedance spectroscopy (EIS). The electrolyte samples were performed in blocking type cells where the DFOB-GPEs were sandwiched between two stainless steel electrodes. The ionic conductivity of the DFOB-GPEs having an area of 1.7665 cm^2^ were measured using ac-impedance spectroscopy (*Ivium Technologies*, Netherlands) in the frequency range (1–10^6^) Hz with amplitude signal of 10 mV. The temperature dependence of ionic conductivity was performed in the temperature range between 298 and 398 K. The ionic conductivity of DFOB-GPEs was calculated using following equation,

4 $$\sigma =t/{R}_{b}A$$

where *t* is the thickness of the DFOB-GPEs, *R*_*b*_ is the bulk resistance and *A* is the area of electrode- electrolyte contact.

The total lithium transference number (*t*_Li_^+^) of prepared DFOB-GPEs was measured using AC impedance and DC polarization method. A step voltage of 10 mV was applied across the symmetrical Li/DFOB-GPE3/Li cell and the resulting current was measured as a function of time (chronoamperometry) at 333 K.

The linear sweep voltammetry and cyclic voltammetry analyses of DFOB-GPEs were examined using Pt/DFOB-GPEs/Li cell at 298 K. These analyses were carried out using *Ivium Technologies* electrochemical workstation. For LSV, the potential range fixed from −5 to +6 V at a scan rate of 20 mV/s. CV analysis was performed at different scan rates in the potential limit between 0 and +5 V. The cycling performance of DFOB-GPE3 was observed in galvanostatic mode using Arbin battery cycler. Charge–discharge analysis was carried out for the 2032 coin cell assembled by sandwiching the DFOB-GPE3 between the commercially available LiCoO_2_ cathode foil and lithium metal anode. The coin cell was cycled between the cut-off voltage of 3.0 and 4.2 V at three different *C*-rates at room temperature.

## Electronic supplementary material

Supplementary Information
