# Supplementary material for: An enhanced electrochemical and cycling properties of novel boronic Ionic liquid based ternary gel polymer electrolytes for rechargeable Li/LiCoO2 cells
Source: Sci Rep. 2017 Sep 11;7:11103. doi: 10.1038/s41598-017-11614-1 (PMC5593839; doi:10.1038/s41598-017-11614-1)
Supplement: Supplementary file 1 — Supplementary Information [file 41598_2017_11614_MOESM1_ESM.doc]

**Electronic Supplementary Information**

**An enhanced electrochemical and cycling properties of novel boronic Ionic liquid based ternary gel polymer electrolytes for rechargeable Li/LiCoO_2_ cells**

**K.Karuppasamy^1*^, Hyun-Seok Kim^1*^, Dongkyu Kim^2^, Dhanasekaran Vikraman^1^, K. Prasanna^3^, A.Kathalingam^4^, Ramakant Sharma^5^, Hee Woo Rhee^2*^**

^1^Division of Electronics and Electrical Engineering, Dongguk University-Seoul, Seoul 04620, South Korea

^2^Polymer Materials Lab, Department of Chemical and Biomolecular Engineering, Sogang University, Seoul, South Korea

^3^Electrochemical Energy Storage and Conversion Lab (EESC), Kyung Hee University, 1732, Deogyeong-daero, Giheung-gu, Yongin, Gyeonggi 17104, South Korea

# ^4^Millimeter-wave Innovation Technology (MINT) Research Center, Dongguk University-Seoul, Seoul 04620, South Korea

# ^5^Plastic Electronics and Energy Laboratory, Department of Metallurgical Engineering and Materials Science, Indian Institute of Technology Bombay, Powai 400 076, Maharastra, India.

***Corresponding authors email: [karuppasamyiitb@gmail.com](mailto:karuppasamyiitb@gmail.com) (K.K), [hyunseokk@dongguk.edu](mailto:hyunseokk@dongguk.edu) (H.-S.K) & [hwrhee@sogang.ac.kr](mailto:hwrhee@sogang.ac.kr) (H.W.R)

**S1 – Characterizations**


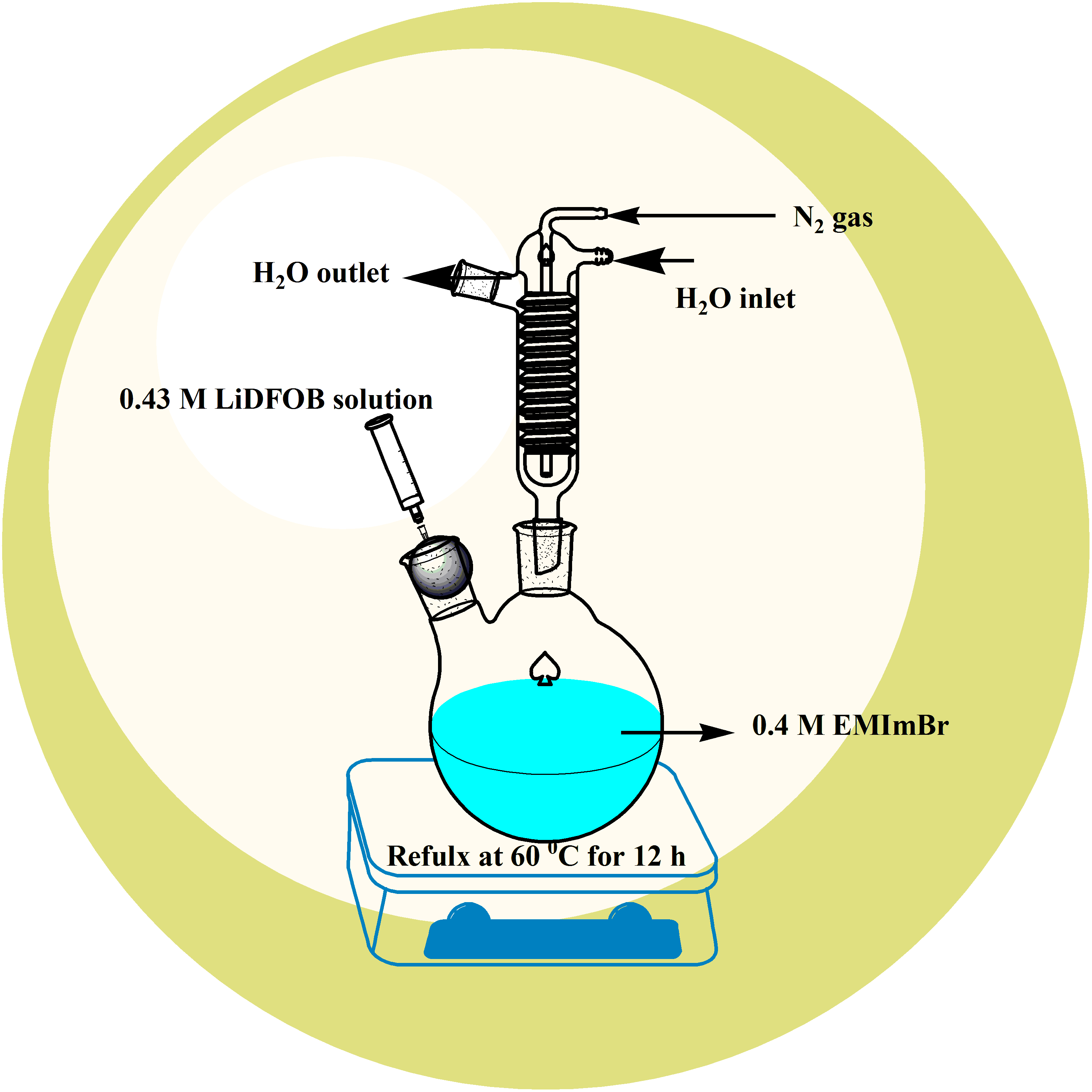


**Fig. S1** Schematic diagram for synthesis of EMImDFOB ionic liquid

**
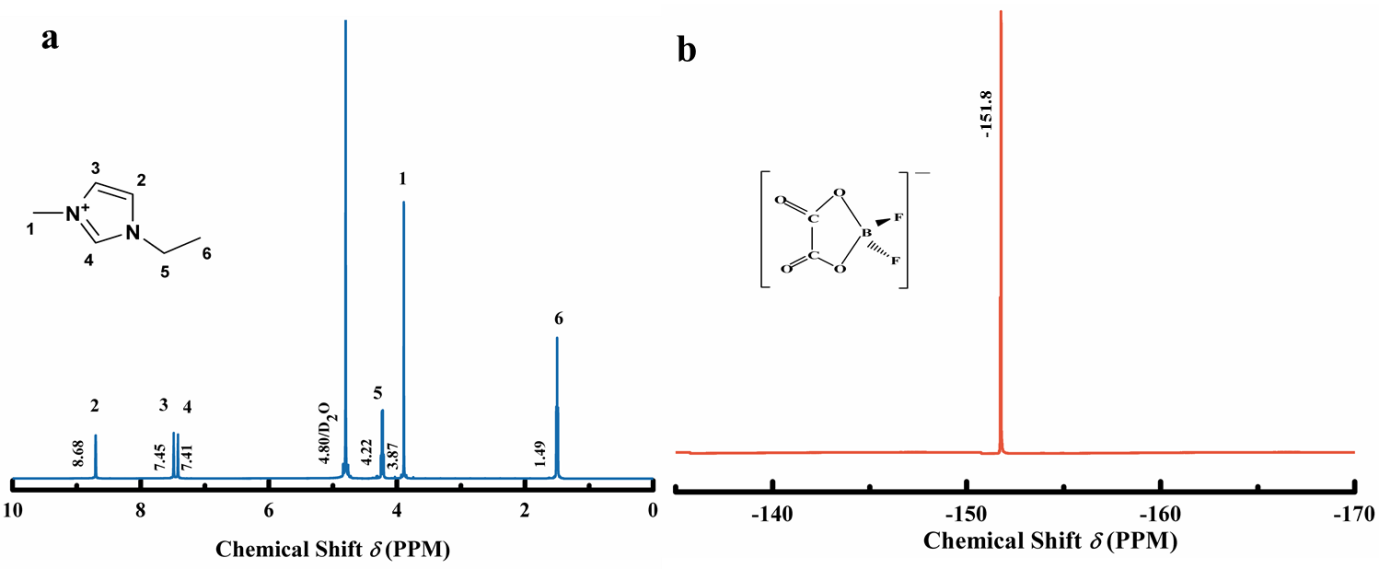
**

**Fig. S2** ^1^H and ^19^F NMR of synthesized EMImDFOB ionic liquid


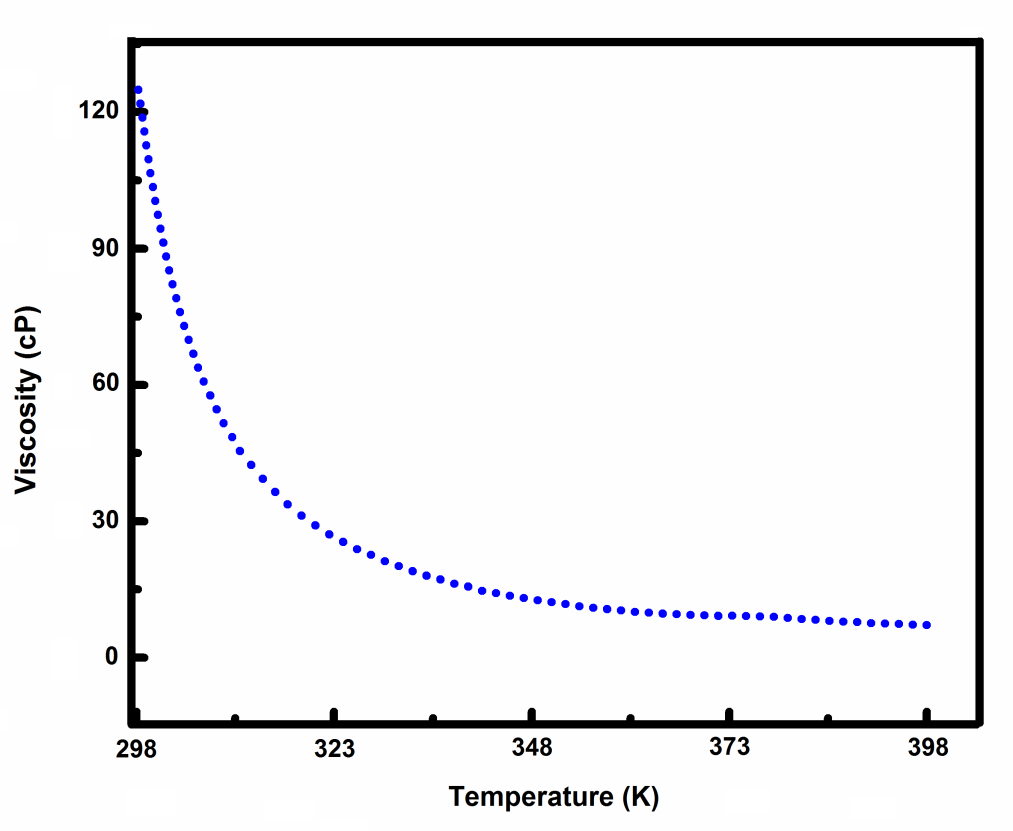


**Fig. S3** Viscosity as a function of Temperature for EMImDFOB ionic liquid


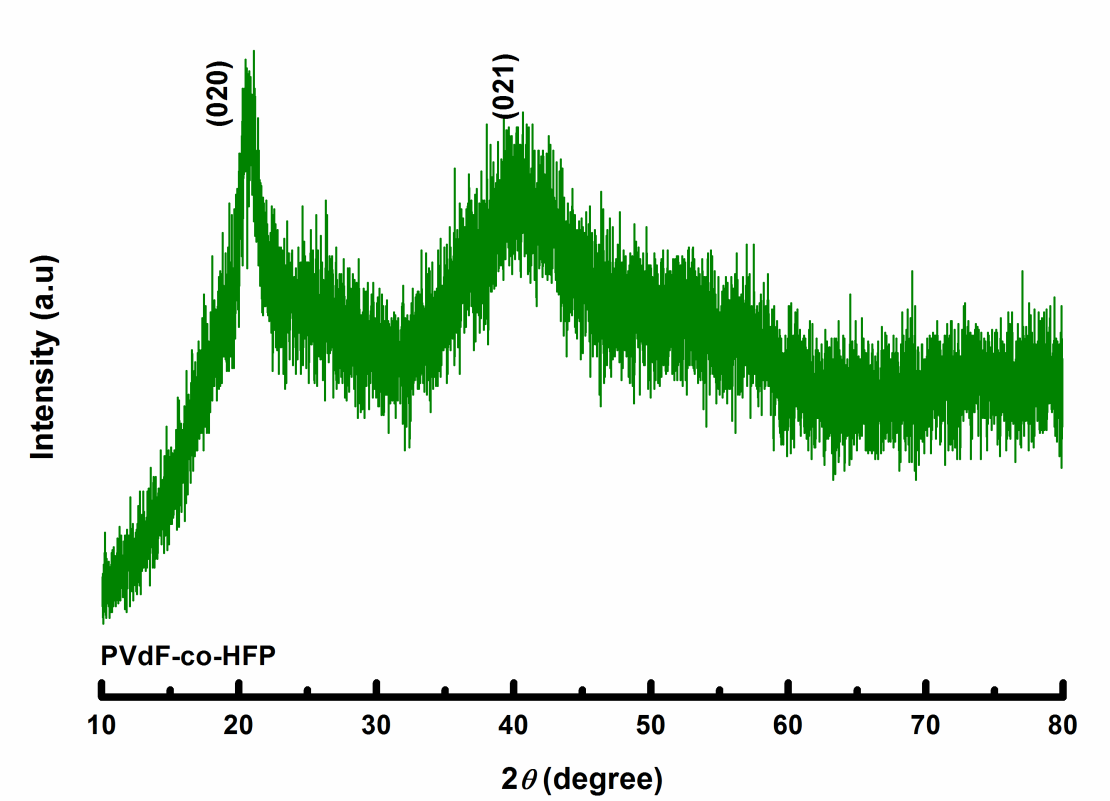


**Fig. S4** XRD pattern of pristine *PVdF-co-HFP*

*
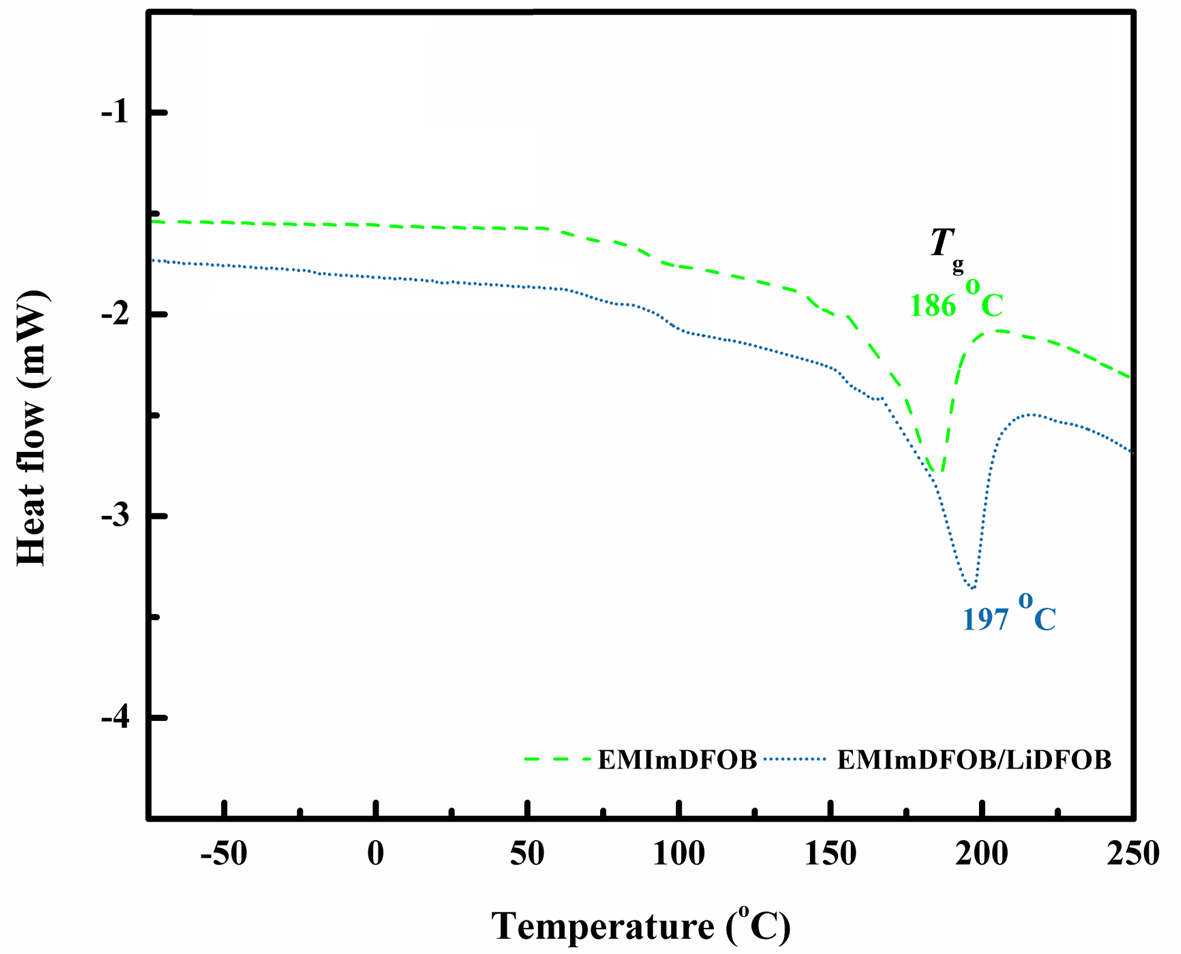
*

**Fig. S5**  DSC thermogram of EMImDFOB and EMIMDFOB/LiDFOB


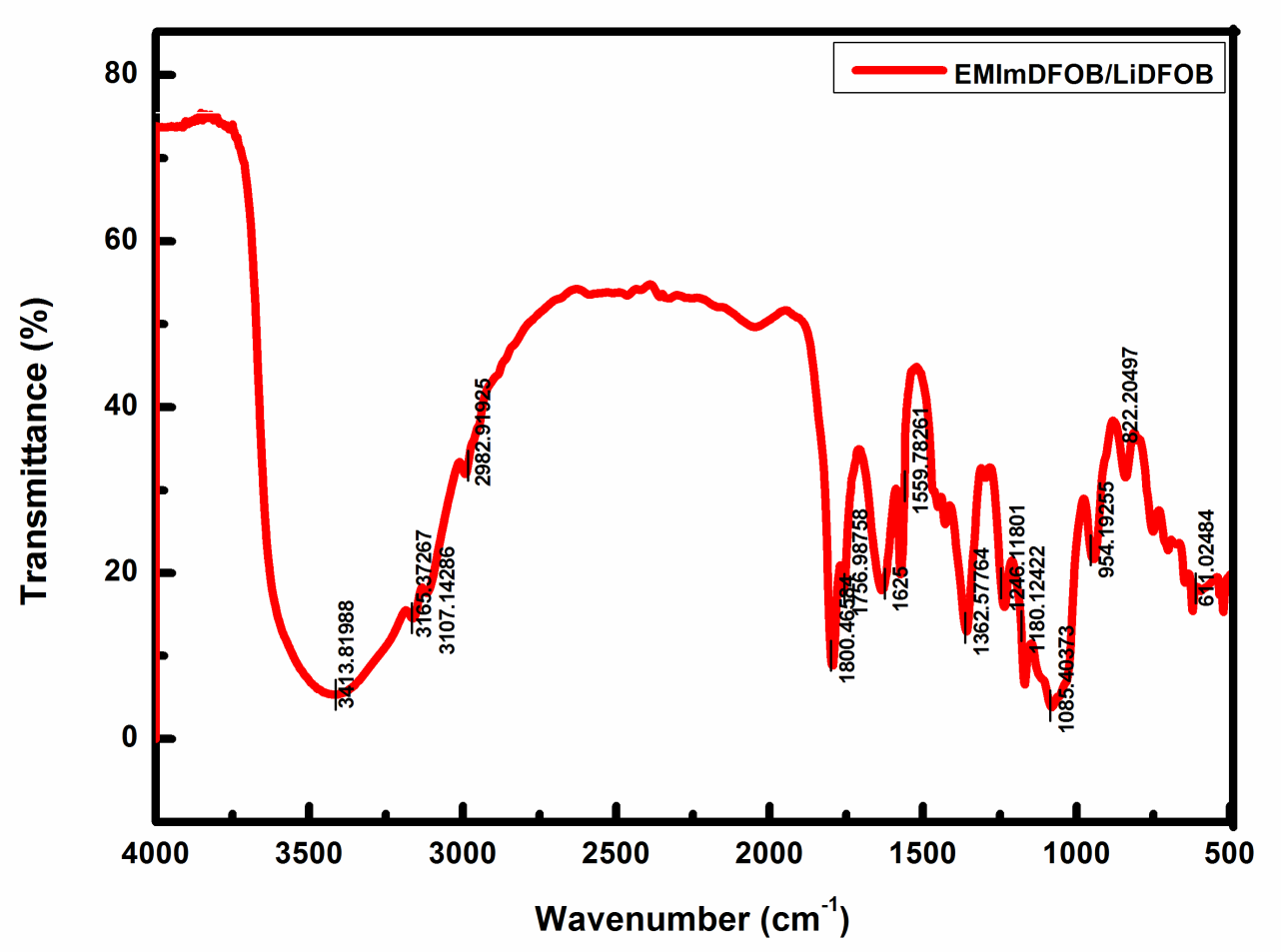


**Fig. S6** FTIR spectrum of electrolyte mixture EMImDFOB/LiDFOB


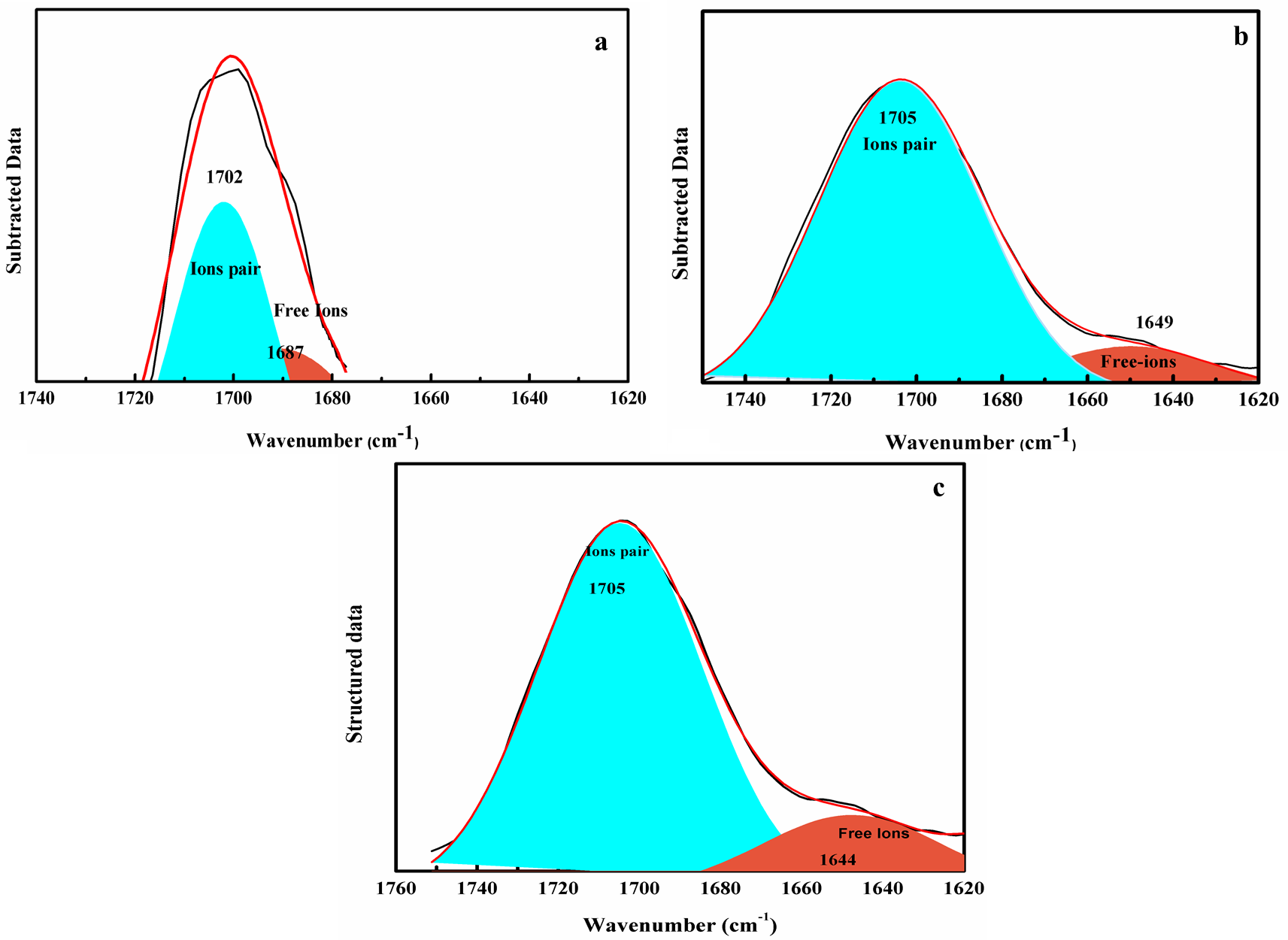


**Fig. S7** Deconvoluted FTIR spectra of (a) DFOB-GPE1 (b) DFOB-GPE2 and (c) DFOB-GPE3 in the wavenumber region between 1740 and 1620 cm^-1^ (Region –I)





**Fig. S8**  TGA thermogram of EMImDFOB and EMIMDFOB/LiDFOB


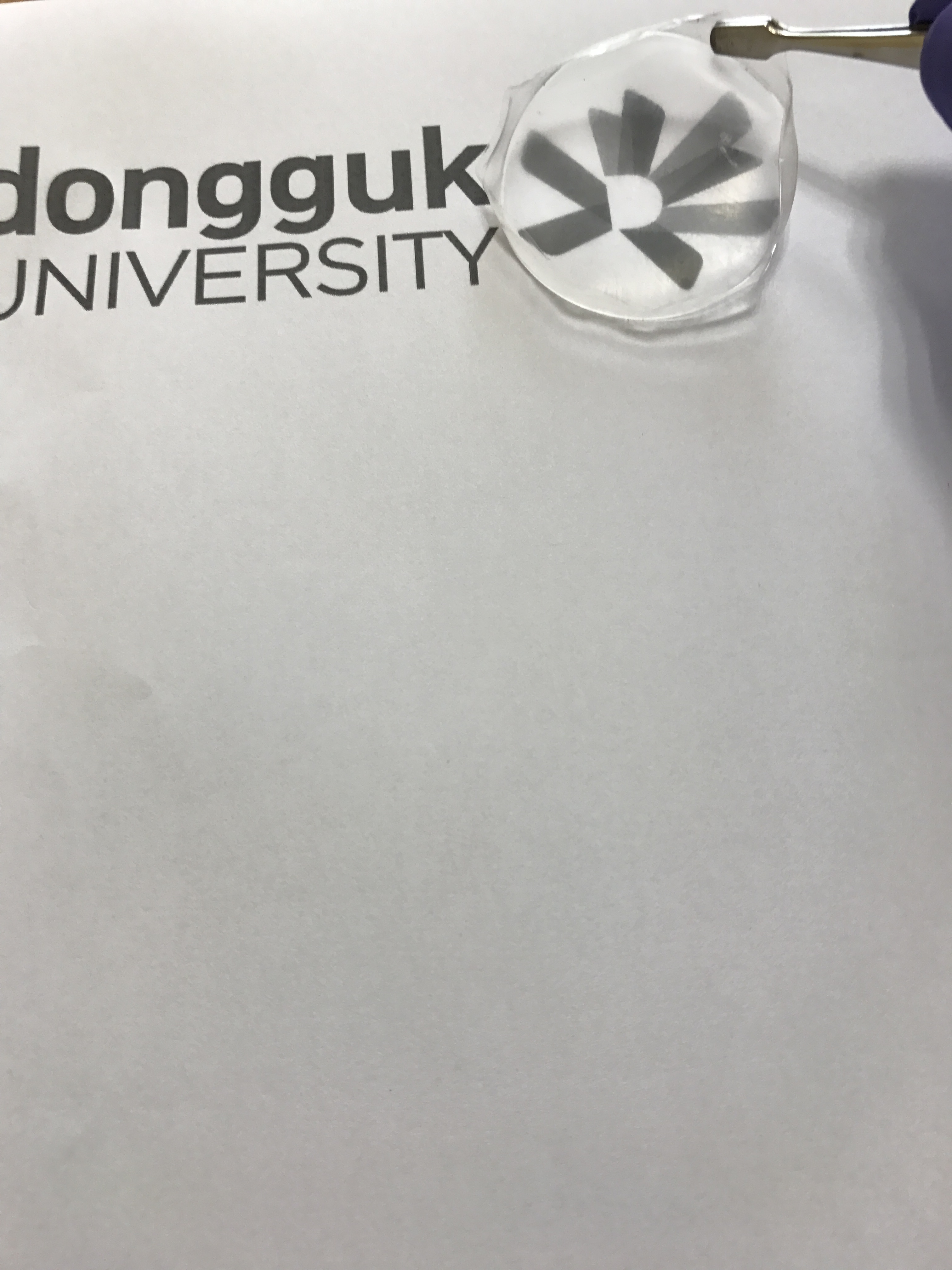


**Fig. S9**  Photo-image of DFOB-GPE membrane

**Table S1**  Equivalent circuit parameter values for DFOB-GPE3 at different temperatures

| **Temperature (K)** | **R1 (Ohm)** | **R2 (Ohm)** | **CPE1-T** |
| --- | --- | --- | --- |
| 298 | 0.0000375 | 3263 | 0.00000058 |
| 318 | 39.0 | 432.3 | 0.00000051 |
| 338 | 75.1 | 98.8 | 0.00000061 |
| 358 | 39.9 | -- | 0.000030 |
| 378 | 28.2 | --- | 0.000021 |
